# The possibility of a human-borne with bioagent (HBBA) terrorist at foreign FOB ECPs: the perceptions of U.S. military or security personnel, a preliminary report

**DOI:** 10.1186/s40779-015-0064-z

**Published:** 2015-12-18

**Authors:** George Edafese Alakpa, John W. Collins Jr

**Affiliations:** Department of Professional Security Studies, New Jersey City University, Jersey City, NJ USA

**Keywords:** Human-borne with bioagent (HBBA) terrorist, Bioterrorism, Combat post ECP

## Abstract

**Background:**

The global war on terrorism has prompted an increase in the deployment of security personnel from multi-national forces on foreign lands, especially in places where known terrorist groups are based. The aim of this study was to obtain U.S. military and security personnel’s perceptions of the possibility of encountering a human-borne with bioagent (HBBA) terrorist at an entry control point (ECP).

**Methods:**

This study was a mixed-method, cross-sectional, survey-based, time-limited study. A validated, five-option Likert scale questionnaire with Cronbach’s alphas of 0.82 and 0.894 for Constructs 1 and 2 was distributed to over 113 respondents with combat experience.

**Results:**

The results indicated that 92.3 % of the respondents thought it was possible for a terrorist to employ a biological agent to cause terror; 61.5 % claimed it was either possible or very possible, and 26.9 % claimed it was somewhat possible for a terrorist carrying a biological agent to successfully breach a combat Forward Operating Bases (FOB) ECP undetected. 26.9 % of the respondents agreed that “ECP soldiers are knowledgeable about bioagents (BA)”, only 15.4 % responded that ECP soldiers have effective devices for detecting a BA on a terrorist at an ECP.

**Conclusion:**

Despite some limitations, this pre-study tends to indicate that while many U.S. military or security personnel acknowledge the possibility of an HBBA terrorist breach and the vulnerability of U.S. combat post ECPs to a BA breach, the soldiers at the ECPs lack adequate knowledge or devices to effectively detect a BA on a terrorist at an ECP.

## Background

Terrorism is defined in numerous ways by different agencies, governments and individuals. The common denominator among the various definitions is that terrorist acts are meant to create public fear and generate publicity for the terrorist’s course. The global community has not been spared from one particular terrorist act: the 2001 World Trade Center attack. This event ignited a global shift in the way the world reacted that has led to a global war on terrorism. The United States military, in its duty to execute the nation’s security strategies, has deployed service men and women into combat and thus has dispersed widely throughout the world. According to the Department of Defense’s (DoD’s) Base Structure Report (BSR), as of 2010, there were 662 facilities maintained by the U.S. Military in 38 foreign countries, excluding those in Iraq and Afghanistan [1]. The exact number of military personnel and the numbers of U.S. military bases on foreign soil, especially combat bases, is unknown to civil society. An electronic search reveals numerous assumptions about the accurate size of the U.S. military. An article by Daniel R. Cobb claimed that in 2009, the “Pentagon acknowledge maintaining 865 active U.S. military bases in 130 countries outside the U.S.”, not including bases in Iraq or Afghanistan ([2], pp. 1–2).

### Bioterrorism

Bioterrorism (BT) simply means an act performed by a terrorist that uses a microbiological agent (bioagent) as a means to create fear and panic in a community. Ashford et al. [3] defined BT as the “intentional use of microorganisms or toxins derived from living organisms to cause death or disease in humans, animals, or plants on which we depend” [p. 515]. Microorganisms such as many of those employed as bioweapons are ubiquitous; they are widely found in nature, and many could be intentionally genetically modified to increase their capability to inflict severe damage or disease [4].

The use of a biological agent (BA), which can be a microorganism or the product of a microorganism (such as a toxin), in biological warfare is ancient, dating back to the 4^th^ or 6^th^ centuries BC [5–7]. The advances delivery of BAs commenced in the 14^th^ century, when catapults were used to deploy the cadavers of people who died of plagues (*Yersinia pestis*), such as during the siege of Kaffa by the Tarta army in 1346 and by the Russians against the Swedish city of Reval in 1710. During the French and Indian Wars, Sir Jeffery Amherst was reported to have sent the blankets and handkerchiefs of smallpox-stricken dead soldiers to Native Americans who were allied with the French troops. A similar technique was reportedly employed by Francisco Pizzarro in his campaign against the natives of what is now Peru during the 16^th^ century [4, 5, 7].

The first known BT in the U.S. is reportedly the 1984 contamination of an Oregon salad bar contamination by a “Bagwan Shree Rajneesh” religious cult group. In 1996, a *Shigella dysenteriae* type2 agent was used to contaminate muffins and donuts in Dallas, Texas. In Washington, DC, and Los Angeles, anthrax hoaxes were reported in 1997 and 1998, respectively; an actual anthrax attack occurred in the widely remembered 2001 October postal contamination incident [7–9]. Tucker [10] reported that of 415 incidents between 1960 and 1998 in the public domain (incidents in the classified domain were excluded) that involved chemical, biological, radiological or nuclear material (CBRN), 151 were terrorist events; 33 of these involved the use of biological agents. Other bioterrorist events during this period, as reported by Tucker [10] and Dudley [11], included the use of eight microbial pathogens, including typhoid fever, diphtheria, dysentery and meningitis, the Salmonella bacterium or *Francisella tularensis* here in the homeland.

After the attacks on September 11, 2001, the U.S. Homeland Security Department received documented reports of anthrax spore exposure, including 11 inhalational cases, 11 cases of cutaneous anthrax, and five deaths [9]. On March 2002 in Texas, the 12th cutaneous anthrax case was reportedly detected and was linked to mail in a Texas laboratory. In 2003, a total of nine ricin BA threats were reported [9], and the ricin toxin was discovered in a South Carolina postal facility in October 2003. On February 3, 2004, the Dirksen Senate Office Building in Washington, DC, was reported to have discovered ricin in the office of Senator Bill Frist. As recently as April 2013, letters that tested positive for ricin were reportedly sent to Senator Roger Wicker [12], and similar letters were reportedly sent to United States President Barack Obama and then-mayor of New York City, Michael Bloomberg [13].

### Terrorist desire to obtain bioagents

Martin et al. [14] documented that the Al Qaeda group “initiated a biological weapon program in Afghanistan before the overthrow of the Taliban regime” [p. 14], and the U.S. military uncovered two of the laboratories that had commercially supplied microbiological bioagents in 2001. Additionally, in 2003, the U.S. forces operating in the north of Iraq seized a camp linked to the terrorist group and found equipment and instructions for ricin extraction [14]. Other documents indicating the extreme terrorist groups’ desires to acquire and utilize BAs, especially against U.S. interests, are reported in [8, 15, 16].

### Exposure of soldiers to BA

While many studies have examined the possible exposure of deployed soldiers to infectious agents [17–19], little is known or available for public/academic review regarding the effectiveness of the U.S. combat Forward Operating Bases’ (FOBs’) protective protocols against terrorists with a BA at the ECP (Entry Control Point) or about the perception(s) of personnel about bioterrorism. This information is significant, especially because most combat FOBs are situated in countries with terrorist groups that are hostile towards or are actively battling U.S. military forces. It is hoped that this information will provide leadership and management ideas for modifying security and training policies to better prepare and educate military personnel to deter, detect and degrade any bioterrorism threat. Moreso, this information will provide insight into the level of understanding or education and will examine whether bioterrorism preparedness is adequate, especially when most FOBs employ numerous local foreign nations to work in these FOBs while they reside outside the FOBs.

### Statement of problem

During combat duties in Afghanistan, one of the authors observed and detected more than one Afghan local national (LN) with certain infectious skin conditions working in the DIFACs (dining facilities) of major FOBs, serving food to soldiers inside the base. These LNs resided outside the FOB and gain entrance to the FOB daily, passing through security parameters established by the DoD. There are tactics, techniques, and procedures (TTP) to prevent or mitigate person-borne improvised explosive devices (PBIED) and vehicle-borne improvised explosive devices (VBIED) and to respond to or recover from chemical, biological, radiological and nuclear (CBRN) attacks on FOBs.

The authors are unaware of any study to date that has examined the perceptions of current or former combat or security personnel regarding the possibility of an HBBA terrorist’s attempt to breach a combat base’s entry control point or how much these personnel know about bioterrorism and/or bioagents.

### Method

This study was a mixed-method, cross-sectional, survey-based, time-limited study. It employed the distribution of a validated five-Likert-Scale-type questionnaire with Cronbach’s alphas of 0.82 and 0.894 for Constructs 1 and 2 [20].

The respondents were military or security personnel with Anti-Terrorism (AT) Tactics, Technique and Procedure (TTP) experience and the ability to complete a questionnaire comprising approximately 42 questions or three quarter of the questions.

Over 113 questionnaires were disseminated between April and August of 2014 with letters of introduction and consent forms. The questionnaires were distributed through the points of contact (POCs) for installations, military units, and military school coordinators, and in some cases, the documents were sent directly to military personnel who showed willingness to participant, most of whom were deployed or returning from recent combat deployment. The completed questionnaires were returned via POCs who either mailed them in sealed packages or emailed them in bulk, or they were individually mailed back to researcher.

The data collected were analyzed using the Statistical Product and Service Solution (SPSS) statistical software (Base Grad Pack shrink wrap version 21.0) for both descriptive and scale reliability (Cronbach’s alpha analysis).

### Ethical statement/approval

The New Jersey City University IRB approved this research on 05/13/2014 as part of the corresponding authors’ DSc dissertation. The respondents’ privacy was protected, and no identifying personal information was collected.

## Results and discussion

Only 26 questionnaires met the established criteria and constituted the sample for this preliminary study. Many of the excluded questionnaires were from respondents who lacked personal AT or TTP/ ECP experience, while others had more than 10 unanswered questions.

### Limitations of results

During the dissemination of the survey tool (questionnaire) to the target population, we found that many in military personnel and Customs and Border Control/Immigration Service agents were reluctant to participate or to allow their subordinates to participate. This ultimately affected the response rate, the number of respondents, and eventually the sample size of this pre-test study. The small sample size made it impossible for the researchers to make a broad generalization or inference from the findings of the study. However, it is important to emphasize that the results tend to show that the ECPs of combat FOBs are vulnerable to breach by a terrorist carrying biological agents. Additionally, these perceptions come from people who have been recently deployed (for the most part) and have ECP TTP experiences in a combat environment. Over 92 % of the respondents in this study whose questionnaires were completed, returned and analyzed were combat veterans with a minimum of two tours of deployment, and with personal ECP TTP experience.

### Descriptive data of the respondents

Slightly more than eighty percent (80.8 %) of respondents whose questionnaire were selected and analyzed identified the military as their profession; 15.4 % were retired military, and 3.8 % were from the Department of Homeland Security. In terms of the military service branch, 80.0 % were in the U.S. Army, 12.0 % were in the U.S. Air Force, 4.0 % were in the U.S. Navy, and 4.0 % were in the U.S. Marine Corps. Of this sample, 69.2 % were enlisted, 26.9 % were officers and 3.8 % were civilians.

### Combat experience of the respondents

More than ninety-two percent (92.3 %) of the respondents had been deployed to combat zones. In terms of the combat campaign in which they participated, many had multiple deployments to more than just one campaigns, with 75.0 % just returning from the Operation Enduring Freedom (OEF) campaign in Afghanistan, 16.7 % just returning from the Iraq war (Operation Iraqi Freedom-OIF), and 8.3 % involved in the Operation New Dawn (OND) campaign. All of the respondents claimed to have had personal knowledge of or training in anti-terrorism procedures.

#### Respondents’ perceptions of the possibility of a biological agent being used as weapon for terrorism and BA TTP training and deployment

**Fig. 1:**
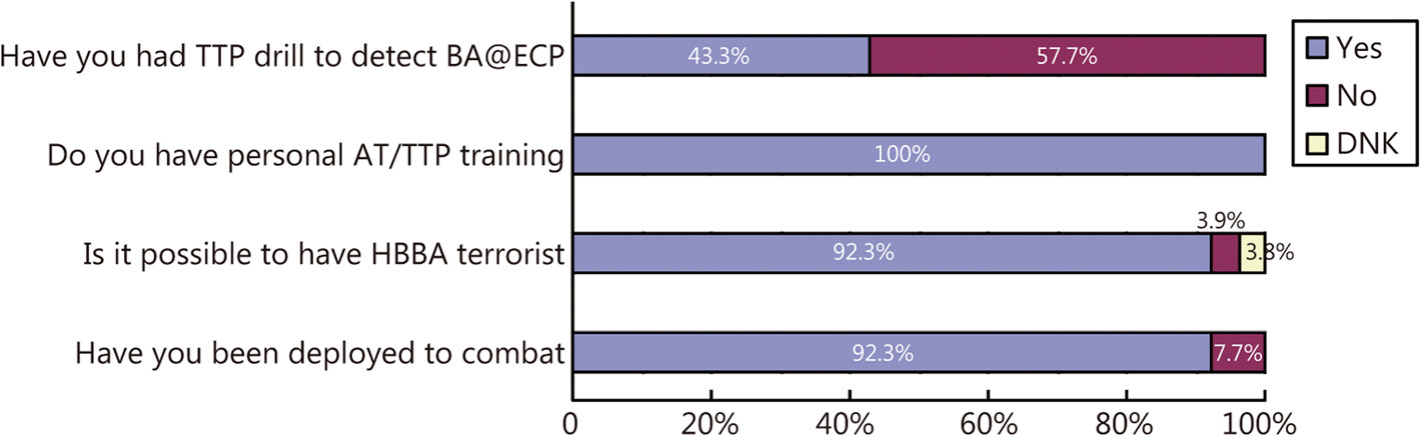
Respondents’ perceptions and their distribution

In response to the questionnaire item asking whether a human-borne with bioagent (HBBA) terrorist attack was possible, 92.3 % of the respondents answered “Yes”, it is possible for a terrorist to employ a biological agent to cause terror. This high positive response may have occurred because the majority of the respondents had been deployed to combat zones and had had anti-terrorism training. However, less than 50.0 % (43.3 %) of the respondents had undergone TTP drills meant to prevent or detect bioagents at the ECPs (Fig. 1).

#### What is the possibility that a terrorist carrying a biological agent will successfully breach a combat ECP undetected?

**Fig. 2:**
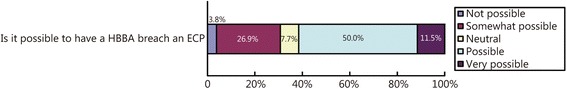
Number of respondents (%) that believed in the possibility of a successful breach of the ECP with a BA

Slightly more than sixty-one percent (61.5 %) of the respondents reported believing that it is possible or very possible for a terrorist carrying a biological agent to successfully breach a combat FOB ECP undetected (Fig. 2).

#### Respondents’ knowledge level regarding BA and possession of BA-detecting device while on duty at an ECP

**Fig. 3:**
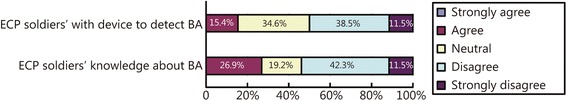
Soldiers at ECPs’ knowledge about BA and their possession of effective BA detection devices

An analysis of ECP soldiers’ knowledge about bioagents and whether they have devices that can effectively detect traces of bioagents on a person indicated that 53.8 % of respondents disagreed or strongly disagreed that soldiers at the ECP are adequately knowledgeable about how or what to look for in terms of bioagents (Fig. 3). In terms of whether every soldier at the ECP has devices that can effectively detect traces of a bioagent borne on a person, 50.0 % of respondents either strongly disagreed or disagreed that soldiers at ECPs have such devices (Fig. 3).

The vulnerability of military personnel to microbial agents during deployments have been documented in many studies [19, 21–23]. Vento et al. [22] reported an increase in the isolation of resistant organisms from the wounds of injured combat service members; multidrug resistant (MDR) *Escherichia coli* (*E. coli*) agents were isolated from the wounds of soldiers deployed to Afghanistan, which resulted in the dissemination of this MDR *E. coli* strain from Afghanistan into the U.S.A.

The results of this pre-study tend to indicate that the majority (61.5 %) of the respondents believed that the time of this study and based on the TTPs, the ECPs of U.S. combat post are vulnerable to a successful breach by a terrorist with a bioagent. This finding is similar to a previous study conducted in Lagos, Nigeria [24], where 64.3 % of security personnel at the airport’s port of entry (POE) had similar thoughts. Additionally, that study reported that 87 % of the security officers believed that an HBBA attack was possible, similar to the 92.3 % of respondents in this study who reported the same belief.

If PBIEDs and natural human carriers of pathogens are possible, it is only plausible to infer that the possibility of humans intentionally incubating BAs with a suicidal terrorist intention. Researchers call such carriers “human-borne with bioagent” (HB-BA) suicide terrorists. In the instance of a vulnerability of current global metal or personal body search procedures (which are specific to explosives), terrorists can also purposely transport a BA in a culture medium in an innocuous potable container (A culture is a medium [abiotic or biotic] for propagating [growing] microorganisms).

The fact that at the time that this report was written, there has been no reported BT breach of any U. S. forward operating combat base is no indication that such an attack could not occur; there is always a first time, as in the case of the September 11, 2001 airplane attacks. As noted in the introduction, BT incidences have occurred in the U.S. in the past, and Hylton [25] documents a breach of White House security with a modified anthrax bacillus.

As in all past pandemic or endemic incidents, humans have been involved in the transborder dissemination of pathogens globally, via travel, and this method could be exploited by terrorists. Galamas [8] referred to people who carry infectious BAs on themselves purely for harmful purposes “suicide bioterrorists, who are less bothered about the need for bio-secure facilities” (p. 85) who “can thus transport BAs under untraceable BTs as a dissemination mechanism that will provide greater advantage to Al Qaeda’s operatives” [p. 85].

While acknowledging the limitations of this study, the authors are unaware of any similar specific study of this group of respondents focusing on combat post ECPs. While the findings of this study are preliminary, they tend to indicate a possibility of BA vulnerability at the ECPs of combat bases and suggest that the majority of soldiers stationed at ECPs lack adequate knowledge about BAs or devices to detect or deter a possible terrorist with a BA at the ECP. How would officers of the Customs and Border Protection department perceive this issue, and how well educated are they regarding BAs identification at the various nations’ POEs? These are questions that can only be answered with another large, extensive study including this group of security personnel, who were not allowed to participate in the present study. On September 30, 2014, the country reported its first indexed case of human-imported Ebola virus in a passenger flying in from Liberia who was already incubating the virus as he passed undetected through a U.S. airport POE. On that day, the nation was informed by Center for Disease Control and Prevention (CDC) officials that the country had confirmed its first Ebola case in Texas [26]. This case illustrates the feasibility of a BA passing through an ECP, even in the homeland.

## Conclusion

There is a need for more studies to determine best practices for ECP TTPs’ or POEs’ to improve bioagent detection, deterrence and response in this era of a global war on terrorism, given the ease with which bioagents can be acquired in public markets and the educational level and abundance of individuals who are willing to commit suicide for martyrdom. Despite its limitations, this pre-study tends to indicate that while a large percentage of some U.S. military and security personnel acknowledge the possibility of an HBBA terrorist attack and the vulnerability of U.S. combat post ECPs to a BA breach, soldiers at ECPs lack adequate knowledge or devices to effectively detect a BA on a terrorist at an ECP.

## Abbreviations

HBBA: Human-borne with bioagent
ECP: Entry control point
BA: Biological agent/bioagent
DoD: Department of defense
BT: Bioterrorism
CBRN: Chemical, biological, radiological, nuclear
FOB: Forward operating base
DIFAC: Dining facility
LN: Local national
TTP: Tactics, techniques, procedures
PBIED: Person-borne improvised explosive device
VBIED: Vehicle-borne improvised explosive device
AT: Anti-terrorism
OEF: Operation enduring freedom
